# Isocitrate dehydrogenases in physiology and cancer: biochemical and molecular insight

**DOI:** 10.1186/s13578-017-0165-3

**Published:** 2017-08-03

**Authors:** Hamoud Al-Khallaf

**Affiliations:** Department of Pathology and Laboratory Medicine, King Fahad Specialist Hospital, 6830 Ammar Bin Thabit St, Al Muraikabat, Dammam, 32253 Saudi Arabia

**Keywords:** Cancer, Glioma, AML, Dominant negative effect, Mutual exclusivity, Driver mutations, IDH, Metabolism, Targeted therapy

## Abstract

Isocitrate dehydrogenases play important roles in cellular metabolism and cancer. This review will discuss how the roles of isoforms 1 and 2 in normal cell and cancer metabolism are distinct from those of isoform 3. It will also explain why, unlike 1 and 2, mutations in isoform 3 in tumor are not likely to be driver ones. A model explaining two important features of isocitrate dehydrogenases 1 and 2 mutations, their dominant negative effect and their mutual exclusivity, will be provided. The importance of targeting these mutations and the possibility of augmenting such therapy by targeting other cancer-related pathways will also be discussed.

## Background

Isocitrate dehydrogenases (IDHs) play important roles in cellular metabolism. The roles of NADP-dependent IDH1 and 2 in normal cell and cancer metabolism are distinct from those of NAD-dependent IDH3. IDH1 and 2 gain of a new function (i.e. neomorphic allele) rather than loss of tumor suppressor one, is suggested by the accumulating evidence. The reverse reaction catalyzed by neomorphic IDH1 and 2 alleles is the one that produces (d) 2-hydroxyglutarate ((D)-2HG). The carcinogenic effect of neomorphic IDH1 and 2 alleles can be attributed to the increased intracellular production of this oncometabolite and its detrimental effect on several key enzymes important in cellular growth and differentiation. This article will discuss why, unlike IDH1 and 2, mutations in IDH3 in tumor are not likely to be driver ones. A model will be provided in this article that explains two important features of neomorphic IDH1 and 2 alleles, their dominant effect and their mutual exclusivity. How IDH1 and 2 neomorphic alleles are common in specific tumor types and the possible mechanism behind that will be discussed, as well. It will also explain why it is likely that, in certain tumors, mutations producing these neomorphic alleles are early events, emphasizing the importance of targeting these driver mutations. The possibility of augmenting the effect of such therapy by targeting other cancer-related pathways will also be addressed.

## Genes, protein structure, and function

Five genes encoding human IDHs have been identified. In the cytosol and peroxisomes, IDH1, encoded by *IDH1* gene on 2q33.3 catalyzes the oxidative decarboxylation of isocitrate (ICT) to 2-ketoglutarate (2KG) (also called α-ketoglutarate) to generate NADPH from NADP+ and the reverse reaction i.e. reductive carboxylation of 2KG to ICT that oxidizes NADPH to NADP+. IDH2 encoded by *IDH2* gene on 15q26.1 catalyzes the same reversible reaction within mitochondria [1]. Both IDH1 and 2 function as homodimers and have high degree of sequence and structural similarity between them [2–6]. Both isoforms are known to play important roles, through their forward oxidative decarboxylation reaction, in cellular defense against oxidative damage [7–9] and reductive synthesis as a source of NADPH and in regulating dioxygenase enzymes function by producing 2KG that these enzymes utilize as a cosubstrate [10]. Their reverse reductive carboxylation reaction is also important in several cellular processes, including lipogenesis and glycolysis regulation, through ICT synthesis that in turn produces citrate via aconitase enzyme [11, 12].

Unlike IDH1 and 2, IDH3 is NAD-dependent and has well-established role in tricarboxylic acid cycle (TCA) where it catalyzes the irreversible conversion of isocitrate to 2KG while reducing NAD+ to NADH [13]. This isoform is principally regulated by substrate availability, positive and negative allosteric effectors. Calcium, ADP, and citrate activate it, whereas ATP, NADH, and NADPH inhibit its activity [14]. The 2KG produced by IDH3 is further metabolized to succinate, and the NADH is used by the electron transport chain (ETC) to generate ATP [15]. It is a heterotetramer with two α-subunits encoded by *IDH3A* on 15q25.1–q25.2, one β-subunit encoded by *IDH3B* on 20p13, and one γ-subunit encoded by *IDH3G* on Xq28. It is believed that α-subunits are catalytic while β- and γ-subunits are regulatory [13, 16, 17].

## Their roles in physiology and cancer

Inside the mitochondria, metabolites can be processed for energy generation usually through the production of reduced electron carriers such as NADH in the TCA cycle. The energy of these electrons carried by the reduced carriers will be utilized by mitochondrial ETC to produce ATP. When mitochondrial NADH/NAD ratio is high (e.g. due to high energy level), some mitochondrial metabolites e.g. citrate will be exported to the cytosol to participate in anabolic processes. IDHs play important roles in both the occurrence and the regulation of these two processes.

**Fig. 1:**
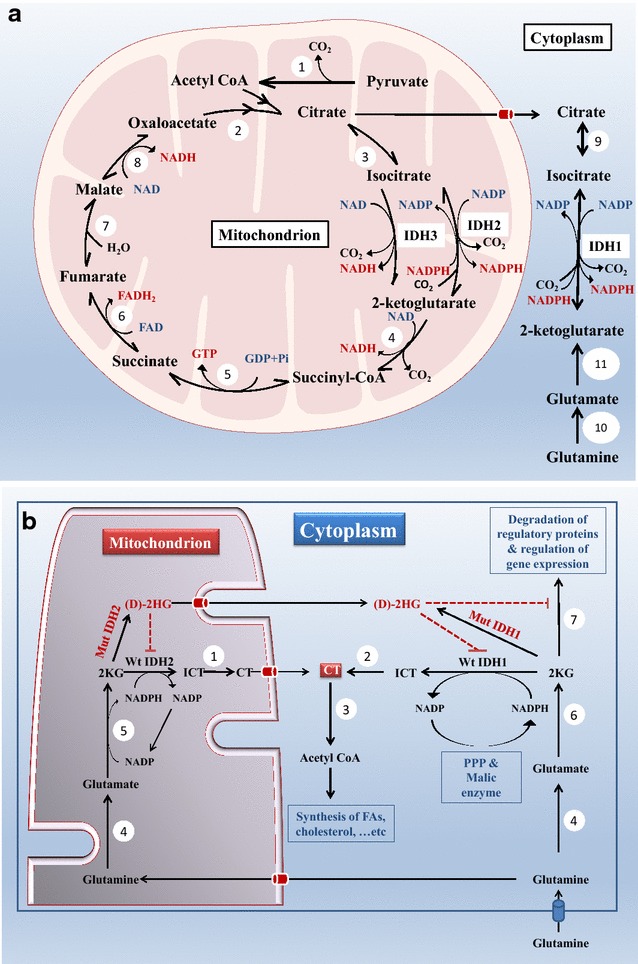
**a** The subcellular localization of the three isocitrate dehydrogenase isoforms and their roles in normal cellular metabolism. *IDH* isocitrate dehydrogenase, *1* pyruvate dehydrogenase, *2* citrate synthase, *3* mitochondrial aconitase, *4* 2-ketoglutarate dehydrogenase, *5* succinyl CoA synthetase, *6* succinate dehydrogenase, *7* fumarate hydratase, *8* malate dehydrogenase, *9* cytosolic aconitase, *10* glutaminase, *11* glutamate transaminase. **b** The roles of wild type and mutant isocitrate dehydrogenases 1 and 2 in cellular metabolism. *CT* citrate, *(D)-2HG* (d) 2-hydroxy glutarate, *FAs* fatty acids, *ICT* isocitrate, *IDH* isocitrate dehydrogenase, *Mut* mutant, *PPP* pentose phosphate pathway, *Wt* wild type, *2KG* 2-keto glutarate, *1* mitochondrial aconitase, *2* cytosolic aconitase, *3* citrate lyase, *4* glutaminase, *5* glutamate dehydrogenase, *6* glutamate transaminase, *7* 2-ketoglutarate dependent dioxygenases. The *dotted red line* indicates inhibition

Due to its large negative free energy change, ICT dehydrogenation to 2KG is one of the irreversible reactions in the TCA cycle. To avoid unnecessary depletion of ICT, this reaction is tightly regulated. It is catalyzed by IDH3 that, in contrast to IDH1 and 2, has extra regulatory subunits. IDH3 is stimulated by substrate availability, e.g. ICT and NAD, and inhibited by ATP and its products i.e. 2KG and NADH [18]. When NADH is high due to high mitochondrial energy level and/or 2KG is high due to its high production from glutamate and glutamine (or due to the high mitochondrial NADH/NAD ratio that in turn inhibits 2KG dehydrogenase complex), mitochondrial ICT level will increase due to IDH3 inhibition. Some ICT molecules will be acted upon by IDH2, the NADP+ dependent mitochondrial isoforms, to produce NADPH, which is utilized to reduce glutathione that participates in the defense against reactive oxygen species (ROS) and repair of mitochondrial oxidative damage [19, 20]. The other ICT molecules will be converted back to citrate by the reversible enzyme mitochondrial aconitase. The accumulating citrate will exit to the cytosol where it produces cytosolic acetyl-CoA by citrate lyase enzyme [21, 22] and NADPH by the cytosolic NADP+ dependent IDH1 [23]. Both NADPH and acetyl-CoA are important for anabolic pathways including fatty acids (FAs) and cholesterol synthesis, emphasizing the important role of citrate in cellular anabolism (Fig. 1a) [24].

In short; in a normal cell, IDH1 and 2 participate in synthetic pathways when the cellular energy level is high enough to inhibit IDH3 and to shuttle citrate out of mitochondria instead of utilizing it by TCA cycle.

The reverse reductive carboxylation reaction of IDH1 and 2, often overlooked, is as important to the cell as the forward oxidative decarboxylation one. In the reverse reductive carboxylation reaction, citrate is formed from ICT produced within mitochondria by IDH2 and in the cytosol by IDH1 by reducing 2KG and incorporating CO_2_ in its structure. Mitochondrial transhydrogenase enzymes that catalyze mitochondrial NADH and NADPH interconversion supply the required NADPH needed for this reaction from mitochondrial NADH that would be abundant in high energy level state. In the cytoplasm, NAPH is mainly supplied by pentose phosphate pathway and Malic enzyme (i.e. NADP-dependent malate dehydrogenase) [25, 26]. The directionality of the reactions catalyzed by IDH1 and 2 largely depends on the relative Km values of the forward oxidative decarboxylation and the reverse reductive carboxylation reactions and the relative levels of ICT and 2KG within the cell [27].

Interestingly, in tumor where the anabolic process is maximized (i.e. lipid synthesize, in particular) [28, 29] and where mitochondrial respiration is slowed down either due to respiratory complexes damage or due to relative inadequacy of oxygen supply to the tumor cell that occurs when the tumor outgrows its vascular supply, the high intramitochondrial NADH/NAD ratio will stop TCA cycle [30, 31]. That in turn, will shuttle the accumulating citrate molecules (some of which were produced by the IDH2-catalyzed reductive carboxylation) to the cytoplasm where it acts as an important anabolic molecule for the tumor cell to synthesize the necessary FAs, phosphoglycerides and cholesterol needed for the rapidly dividing cell to synthesize biological membranes [11]. Another known pathway where tumor cells maximize citrate synthesis, is through the excessive utilization of glutamine and glutamate to produce 2KG [32–36], an indirect precursor of citrate through the reductive carboxylation reaction catalyzed by IDH2 within the mitochondria and IDH1 within the cytosol (Fig. 1b) [31, 37–39]. Interestingly, Isotope-labeling experiments on whole cells using uniformly labeled ^13^C-glutamine as a culture media nutrient demonstrated that the carbons in (D)-2HG (i.e. produced by neomorphic IDH1 and 2 alleles, as will be discussed later) are derived from glutamine, with reasonably high overall pathway flux from glutamine through glutamate and 2KG to (D)-2HG [40].

From what has been mentioned so far, it is clear that both the forward oxidative decarboxylation and reverse reductive carboxylation reactions of IDH1 and 2 are not only important to normal cells, but to cancerous cells, as well. As will be elaborated on below, the reverse reductive carboxylation reaction is more active in certain normal cell types e.g. cells that normally produce high amount of citrate like astrocytes and myeloid cells. If neomorphic IDH1 or 2 was present in such cells, these cells would transform into cancerous ones due to the over production of the oncometabolite (D)-2HG. In such cells, the carcinogenic effect of these neomorphic alleles would be more dramatic and; at least theoretically, therapeutic modalities targeting these neomorphic alleles in these tissue types, would be more effective. Even in a cancerous cell transformed by one of these neomorphic alleles, the forward oxidative decarboxylation reaction catalyzed by the remaining wild-type isoform would still be important for that cell to be able to produce NADPH. In other words, cancerous cell that has neomorphic IDH2, would still need the wild type IDH1 isoform to catalyze the forward oxidative decarboxylation reaction to produce NADPH. The production of the later would still be needed by this cancerous cell to negate its relatively highly produced ROS and for the reductive synthesis of FAs and cholesterol needed by this cancer cell to continue its growth and division. That, as will also be discussed later in this article, explains the mutual exclusivity seen in neomorphic IDH1 and 2 alleles where one tumor cell cannot have both IDH1 and 2 neomorphic alleles.

## Mutant isocitrate dehydrogenases in cancer

### Isoform 3 mutations in tumor

As will be elaborated on in a later section of this article; in certain tumors, monoallelic activating mutations in IDH1 and/or 2 (i.e. neomorphic allele producing mutations) have been documented to be recurrent genetic alterations of high diagnostic and prognostic value [41–43]. On the other hand; although several mutations in each of the three IDH3 subunits have been recovered from different types of tumor samples, none of these mutations were found to be as recurrent as IDH1 and 2 activating mutations. Moreover, neither the significance of these IDH3 mutations nor their carcinogenic roles have been proven. The reason behind that has been speculated to be due the fact that the occurrence of monoallelic IDH1 and IDH2 mutations is more frequent by chance than the biallelic mutations expected for IDH3 [44]. In my opinion; IDH3 mutations, whether mono- or biallelic, are unlikely to be carcinogenic. A more plausible explanation to that is as follows: IDH3, like IDH1 and 2, catalyzes ICT conversion to 2KG. Like IDH1 and 2 mutations (including activating ones as will be explained later), a biallelic mutation in any of the IDH3 subunits is expected to inhibit this reaction (monoallelic inactivating mutation will be harmless and will not inactivate this enzyme due to the enough residual activity contributed by the other normal allele). IDH3 is the main isoform involved in TCA cycle and its reaction is in fact the first oxidative reaction in this important cycle where it produces the first TCA cycle derived reduced electron carrier, NADH. Therefore, inactivating IDH3 enzyme by biallelic mutations will inhibit the whole TCA cycle and mitochondrial ATP production, since there would be no mitochondrial production of reduced electron carrier to undergo oxidative phosphorylation by ETC to produce ATP. Taken together; IDH3 biallelic mutations will have detrimental effect on cellular growth causing the cell to apoptose rather than to overgrow and become cancerous, while monoallelic ones will be tolerated by the cell and will have no carcinogenic role. Another reason is the fact that IDH3, unlike IDH1 or 2, does not catalyze the conversion of 2KG to ICT (the reverse reductive carboxylation reaction), so it is not subject to the gain of function effect seen in monoallelic IDH1 and 2 arginine substitution where, as explained below, the oncometabolite (D)-2HG would be produced from 2KG.

Interestingly; unlike IDH3, inactivation of other TCA enzymes like succinate dehydrogenase (SDH) and fumarate hydratase (FH) have been reported in cancer, including paraganglioma and renal cell carcinomas. SDH is a heterotetramer of nuclear-encoded subunits (A, B, C, and D). This enzyme links TCA cycle to the ETC by oxidizing succinate to fumarate and passing the extracted electrons to the mobile electron carrier, ubiquinone, where the later delivers them to ETC complex III. Mutations in subunits B, C, and D have been associated with tumor more frequently compared to those in subunit A. It is thought that succinate accumulation inhibits, prolyl hydroxylase (PHD), a 2KG-utilizing enzyme, due to the structural similarity between them [45]. Hydroxylation of the angiogenic protein hypoxia-induced factor alfa (Hif1α) is a required step for its degradation. Therefore, it is hypothesized that the Hif1α accumulation in SDH deficiency is due to PHD inhibition by the accumulating succinate. However, there are two arguments against the validity of this hypothesis. One of them is the fact that succinate level will increase due to inactivating mutation in any of the four SDH subunits; however, unlike the other SDH subunits (i.e. B, C, and D), inactivating mutations of subunit A has only been reported in a few tumor cases [46–49]. The other argument is the difference in structure between succinate and 2KG. Although both succinate and 2KG are dicarboxylic acids, the former is one carbon shorter than the later, and the later is a keto acid while the former is not. This difference of structure is another argument against the above hypothesized mechanism. Another hypothesized mechanism linking SDH deficiency to tumor, is the increased ROS production from inactivating mutations in subunits B, C, and D, but not A [50]. Due to the configuration of these subunits and the different electron carriers contained within each of them, subunit A is the one that comes in contact with succinate. It oxidizes it and passes its electrons to its contained electron carrier FAD that will be reduced to FADH. Normally the electrons in FADH will be passed through the other electron carriers (i.e. heme group and iron–sulfur clusters) within the remaining SDH subunits before they are passed to quinone. It is thought that if subunit A is normal, and there are defects in the subunit B, C or D, then the oxidation of succinate proceeds but the shuttling of electrons within the whole SDH complex to ubiquinone is impaired. The electrons “stuck” in FADH will react with O_2_ to form superoxide, a type of ROS [50–52]. ROS are thought to elevate intracellular Hif1α by inhibiting the metalloenzyme, PHD, probably by oxidizing its Fe^2+^ that is critical to its function [52]. This hypothesis; however, does not explain how nonsense and deletion mutations, expected to produce no protein, were also described and linked to tumor. If the later hypothesis were true, only missense mutations producing malfunctioning proteins will be linked to tumor, which is not the case. However, IDH3 inactivating mutations on the other hand are not expected to produce ROS and therefore might not have carcinogenic effect. That is because out of the IDH3 subunits, only α subunit is catalytic. It is the one that comes in contact with ICT, oxidizes it, and passes its electrons to NAD to reduce it to NADH. Just like SDH subunit A, inactivating mutations in IDH3 subunit α might not generate ROS, since no electrons will be captured from its substrate, ICT. Unlike SDH, the other IDH3 subunits are regulatory ones and do not contain electron carriers through which the NADH electrons would pass and therefore their mutations are not likely to trap those electrons where they would potentially participate in ROS formation.

The other TCA enzyme where inactivating mutations of which are linked to cancer is FH. Individuals with germline inactivating mutations in the gene coding for this enzyme, *FH*, are predisposed to hereditary leiomyomatosis and renal clear cell cancer (HLRCC) [53]. Tissue analysis in HLRCC tumors revealed excessive ROS and Hif1α levels in the cytosol and nucleus [54, 55]. Just like in SDH inactivation, it is thought that the high Hif1α is due to ROS mediated PHD inhibition [52]. However, the mechanism by which ROS is elevated in FH inactivation is different. Here, it was found that the accumulating fumarate reacted with the reduced glutathione and depleted it. Since reduced glutathione is an important ROS scavenger, its depletion by the accumulating fumarate leads to increased ROS level and qualified fumarate as an oncometabolite [51]. When comparing IDH3 to FH, it is clear that inactivating the former does not lead to an accumulation of an oncometabolite. The substrate of IDH3 is ICT and the accumulating ICT will be converted to citrate. Neither ICT nor citrate is an oncometabolite.

From what has been mentioned so far, it is safe to say that neither biallelic nor monoallelic IDH3 mutations would be cancerous. However, because of the known aberrant tumor DNA replication and repair, IDH3 mutations are found in tumor as “passenger” ones.

### Oncogenes or tumor suppressor genes?

Although some researchers suggested that IDH1 and 2 act as tumor suppressor genes [56], the accumulating evidence disagrees with that. Unlike SDH and FH loss-of-function mutations of which have been identified in various cancers, R132 and R172 mutations in IDH1 and 2 (the neomorphic allele producing mutations); respectively, do not follow Knudson’s two-hit model of tumor suppressor genes. Almost all reported cases of these mutations have been heterozygous, and inactivating alterations such as frameshifts, deletions, and nonsense mutations have not been observed in *IDH1* or *2* in cancer.

In in vitro transformation assays, it was the expression of neomorphic IDH1 and 2 that promoted the proliferation and inhibited the differentiation of immortalized human cells. Expression of the catalytically inactive form of IDH did not have a similar effect [57–59].

Because of these observations, one can conclude that IDH1 R132 and IDH2 R172 mutations lead to gain of function rather than loss of tumor suppressor one [60, 61].

Interestingly, some researchers hypothesized the tumor suppressor roles of IDH1 and 2 based on their function to produce 2KG that in turn would be utilized by dioxygenases e.g. PHD to inactivate tumorigenic proteins like Hif1α (will be discussed in details in the following sections). This hypothesis is not plausible, since even in cells harboring IDH1 R132 or IDH2 R172 mutations, 2KG will still be produced by other pathways including glutamate transamination and dehydrogenation (Fig. 1b). In fact, the mean level of 2KG was not altered in the IDH1 or 2 mutant acute myeloid leukemia (AML) [62] or glioma cells [40], suggesting that the mutation does not decrease the concentration of this metabolite, a finding that does not support the tumor suppressor role of IDH1 or 2.

The gain of function hypothesis, on the other hand, is more plausible since the oncometabolite (D)-2HG produced would inhibit the utilization of 2KG by those dioxygenases even when the later (i.e. 2KG) is present in a sufficient amount (Fig. 1b).

### How does the newly gained function mediate carcinogenesis?

Researchers who studied the mutated (i.e. neomorphic) IDH1 and 2 found that neither ICT nor 2KG where produced by the mutant form [42, 56, 60, 63]. They discovered that the new function was the production of the oncometabolite, (D)-2HG [40, 64].

Recent evidence suggests that metabolites themselves can be oncogenic by altering cell signaling and blocking cellular differentiation. From what has been mentioned so far, it is very likely that the carcinogenic effect of IDH1 R132 and IDH172 is mediated through the elevated cellular (D)-2HG levels originating from cytoplasm by IDH1 R132 or from mitochondria by IDH2 R172 (where it exits the mitochondria and enters the cytoplasm). Recently, the effects of IDH1 R132 and IDH2 R172 mutations on hematopoietic cell lines were replicated using exogenously applied (D)-2HG, not to mention that IDH inhibitors were found to reduce (D)-2HG production and inhibit the growth of leukemia or glioma cells in a mutant-specific manner, proving that this metabolite mediate IDH1 R132 and IDH2 R172 mutated alleles oncogenicity [58, 65–68].

One of the proposed mechanisms of (D)-2HG carcinogenesis is via its competitive inhibition effect on 2KG dependent dioxygenases, due to the structural similarity it has with 2KG, that hydroxylates key proteins and enzymes. Some of those key proteins, such as Hif1α that is hydroxylated by the dioxygenase PHD, are known to be involved in regulating cellular division [56]. Mammalian cells express >60 dioxygenases that utilize 2KG as a cosubstrate and therefore considered potential target of (D)-2HG inhibition [69, 70]. Altering cell physiology and differentiation by inhibiting key dioxygenases, like myeloid tumor suppressor ten-eleven-translocation 2 (TET2) and jumonji C (JmjC) domain-containing histone demethylases, hence impacting the extent of protein and nucleic acid methylation, is another proposed mechanism [71–74]. Interestingly, studies showed that IDH mutant gliomas (i.e. harboring neomorphic IDH allele) are associated with hypermethylator phenotype [75, 76]. It has also been shown that neomorphic IDH1 and 2, as well as loss of function mutations in TET2 establish a hypermethylation phenotype in leukemia [59, 77]. These studies suggest that IDH1 and 2 neomorphic alleles drive carcinogenesis through increased hypermethylation that, in turn, could silence the expression of genes important for cell differentiation and programmed cellular death such as tumor suppressor genes. It is very likely that (D)-2HG produced by IDH1 R132 or IDH2 R172 alleles (i.e. the neomorphic alleles) established the hypermethylation by inhibiting key demethylators such as TET2 and JmjC domain-containing histone demethylases. Notably, in a study conducted by Xu et al. [78], they provided in vitro and in vivo evidence demonstrating that both 2-hydroxyglutarate enantiomers (elaborated on below) act as competitive antagonists of 2KG to inhibit 2KG-dependent TET2 and histone demethylases with (D)-2HG being significantly less potent than (L)-2HG [78]. It is also important to bring the reader’s attention to the possibility that elevated cellular level of (D)-2HG, by neomorphic IDH-independent mechanism could also be carcinogenic. Phosphoglycerate dehydrogenase (PHGDH), an enzyme involved in the de novo synthesis of serine, has recently been shown to catalyze the NADH-dependent reduction of 2KG to (D)-2HG [79]. Amplification of PHGDH (located on chromosome 1p12) occurs in about 16% of all human cancers, including 6% of breast cancers and 40% of melanomas [80]. Over expression of PHGDH in breast epithelial cells enhances the acquisition of malignant properties, whereas silencing of PHGDH inhibits growth of PHGDH-amplified cells, which is why PHGDH is now considered as a potential therapeutic target in tumors. Although the effects of PHGDH on tumorigenesis have not been yet proven to be mediated through increased intracellular (D)-2HG level, based on what has been mentioned so far, this possibility is still likely.

This metabolite is a five-carbon dicarboxylic acid. Its second carbon is chiral, meaning that it has two enantiomers: (D)-2HG and (L)-2HG (also known as (*R*)-2HG and (*S*)-2HG; respectively). Currently, there is no known physiologic role for either enantiomer in normal metabolism. Both enantiomers are believed to be normal “waste” metabolite originating from mitochondria in a small amount that is detoxified to form 2KG by the two enzymes (D)-2HG (D2HGD) and (L)-2HG (L2HGD) dehydrogenase; respectively [81, 82]. In wild type IDH cells, (D)-2HG is produced from hydroxyacid–oxoacid transhydrogenase that converts γ-hydroxybutyrate to succinic semialdehyde while reducing 2KG to (D)-2HG [83]. The other enantiomer (L)-2HG is generated by the TCA cycle enzyme (l)-malate dehydrogenase that interconverts oxaloacetate and (l)-malate [84].

The (D) enantiomer is the 2-hydroxyglutarate form that is exclusively produced by neomorphic IDH-harboring tumor cells and its high production in these tumor cells has been documented [40, 62, 85]. It is believed that this high production exceeds the detoxifying ability of D2HGD leading to its intracellular accumulation even when the later enzyme is functioning normally [86].

It is worth mentioning here that patients with type I d-hydroxyglutaric aciduria, a recessive disorder caused by germline homozygous or compound heterozygous inactivating mutations in D2HGD, have not been found to have increased rate of malignancies. That is probably due to the relatively low amount of (D)-2HG production in these patients and/or lack of follow up studies on these patients due to their relatively short life span. Patients with type II d-hydroxyglutaric aciduria (D2HGA2), a dominant disorder caused by germline neomorphic IDH2 R140 inheritance, has not been found to have increased incidence of malignancy, either. Although that would be considered a strong argument against the carcinogenic effect of (D)-2HG, it can still be argued that these patients die either during infancy or early childhood, and it is therefore difficult to make definitive conclusions about an association between their high levels of (D)-2HG and long-term susceptibility to cancer. Another explanation could be the requirement for additional cooperating somatic mutations for complete transformation and disease progression. Due to their short life span, these cooperating mutations might not have occurred in these patients. Intriguingly, brain tumors have been described in several (L)-2HG aciduria (i.e. an autosomal recessive disorder caused by homozygous or compound heterozygous inactivating mutations in L2HGD) cohorts from distinct geographical regions [87–91]. That is probably because the inhibitory effect of (L)-2HG on 2KG-dependent dioxygenases is stronger than that of (D)-2HG and therefore the carcinogenic effect of the former enantiomer is more than that of the later. It is also possible that patients with (L)-2HG aciduria live long enough to allow the establishment of the correlation between this disease and the risk of cancer. Interestingly, increased (L)-2HG, due to somatic silencing of its degrading enzyme L2HGD, has been proven to be the carcinogenic metabolite transforming renal cells into clear-cell renal cell carcinoma (ccRCC) and adding *L2HGDH* gene to the continuously expanding tumor suppressor gene list [92–95]. The association between (L)-2HG and ccRCC, the histological tumor type frequent in Von Hippel–Lindau (VHL) syndrome, is not surprising. From what has been mentioned so far, this enantiomer like (D)-2HG inhibits Hif1α hydroxylation, an important step needed for its degradation by VHL-E3 ubiquitin ligase complex [96]. Just like in the case of VHL syndrome where Hif1 accumulates due to lack of VHL protein that participates in degrading it, the oncogenic protein Hif1α will accumulate inside renal cells due to the decrease in its hydroxylation due to (L)-2HG inhibitory effect and transform them into ccRCC.

### The oncometabolite comes from isocitrate or 2-ketoglutarate?

Since the reactions catalyzed by IDH1 and 2 are reversible; theoretically, both enzyme substrates were possible sources of (D)2-HG. However, (D)-2HG was proven to be produced by the reverse reaction from 2KG [40]. This could be, as proposed by some researchers, due to a neomorphic function the enzyme gained due to the substitution of its critical arginine [40, 60]. It is also possible that there is no neomorphic function gained by this substitution and the oncometabolite production is simply a result of partial inactivation of the normal reductive carboxylation reaction where the carboxylation part was affected but not the reductive one.

### The dominant negative effect and the mutual exclusivity

**Fig. 2:**
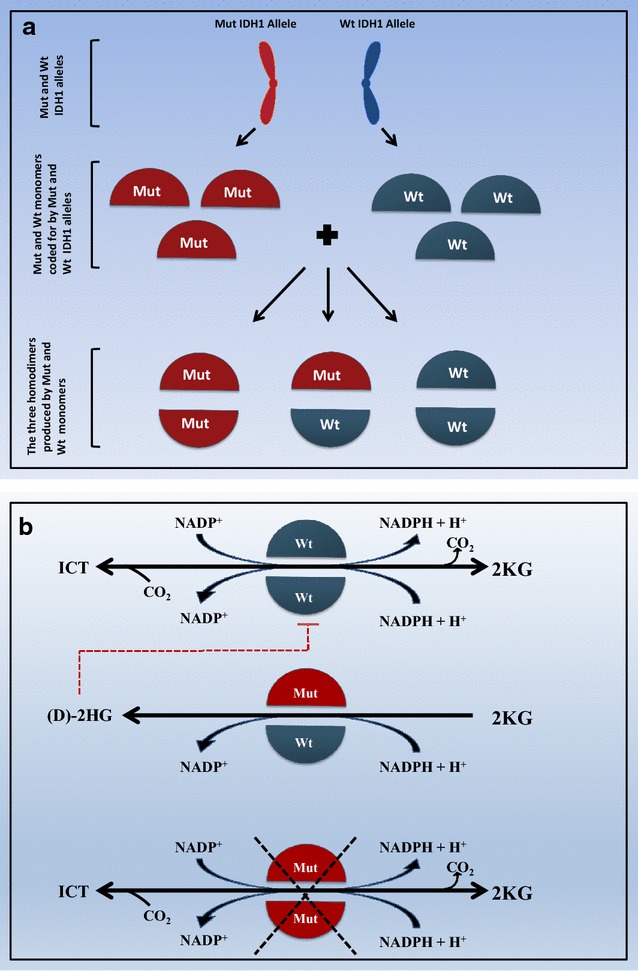
**a** The three dimer types formed in a cancer cell heterozygous for IDH1 mutation. *Mut* mutant, *Wt* wild type. **b** The model gain of function and dominant negative effect exerted by heterozygous IDH1/2 mutation. *(D)-2HG* (d) 2-hydroxy glutarate, *ICT* isocitrate, *Mut* mutant, *Wt* wild type, *2KG* 2-keto glutarate. The *dotted red line* indicates inhibition. The *crossed black lines* indicate loss of enzyme function in both directions

Both IDH1 and 2 are active as homodimers, probably because the active site amino acids (including arginine IDH1 R132 and IDH2 R172) are contributed by both monomers. It is known that in tumor, the recurrent arginine mutation is only found in heterozygous state [97, 98]. The existence of wild type–mutant (Wt-Mut) and mutant–mutant (Mut-Mut) dimers in addition to wild type–wild type (Wt-Wt) dimer in a cell heterozygous for IDH mutation has been proven [56] (Fig. 2a). An illustration of the three dimer types formed in a cell harboring one mutant IDH1 allele is provided in Fig. 2a (i.e. the same applies to IDH2).

When considering ICT oxidative decarboxylation (i.e. forward reaction), heterozygous substitution of R132 in IDH1 with any one of the six amino acids observed in gliomas (i.e. histidine, serine, glycine, cysteine, valine, and leucine) impaired interactions of the enzyme with ICT both sterically and electrostatically. Zhao et al. [56], conducted an in vitro study where they showed that the enzymatic activities of three tumor-derived IDH1 mutants, R132H, R132C and R132S, had a greater than 80% reduction in activity as compared with the wild-type. Similarly, mutation of the arginine residue in pig mitochondrial IDH2 equivalent to R132 in human IDH1 caused a dramatic decrease in ICT oxidative decarboxylation [99]. So, clearly ICT oxidative decarboxylation will only be catalyzed by Wt-Wt, but not Wt-Mut or Mut-Mut dimers. That is probably because the critical arginine has to be contributed by both monomers for the dimer to bind ICT.

When considering the reverse reductive carboxylation reaction, the accumulating evidence supports the production of (D)-2HG by the mutant allele from 2KG. Heterozygous mutations were found to increase this oncometabolite level but not the homozygous one.

**Fig. 3:**
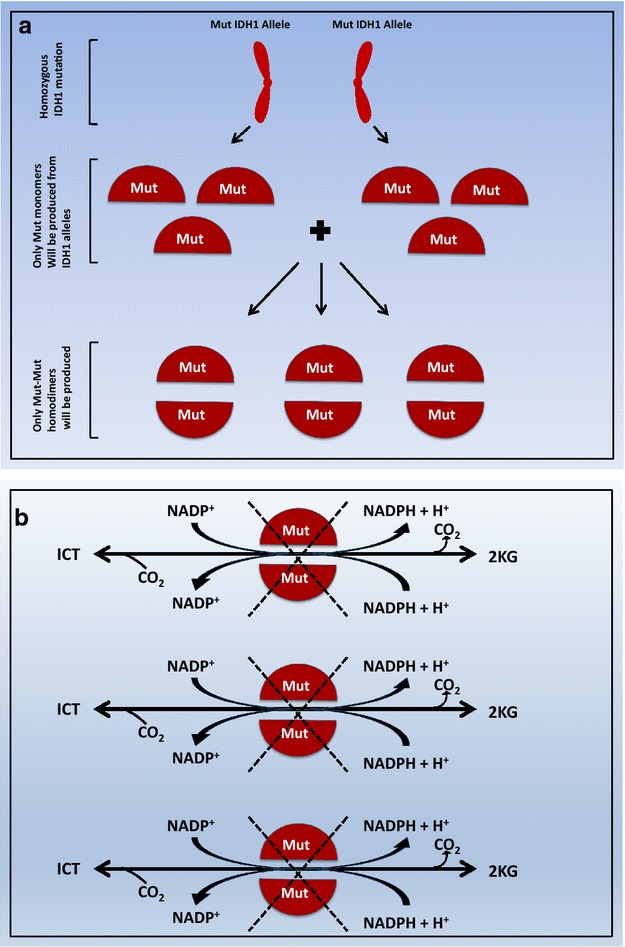
**a** The IDH1 dimer type formed in a cancer cell that is homozygous for IDH1 R132 mutation. *IDH1* isocitrate dehydrogenase isoform 1, *Mut* mutant (e.g. IDH1 R132). **b** The model explains the absence of IDH1 R132 homozygosity in cancer cells. Since only the Mut-Mut homodimer will be produced in the cancer cell that is homozygous for IDH1 R132, no (D)-2HG will be produced and therefore the oncometabolite drive will be lost causing that particular cancer cell to regress and undergo apoptosis. *ICT* isocitrate, *Mut* mutant (e.g. IDH1 R132), *Wt* wild type, *2KG* 2-keto glutarate. The *dotted red line* indicates inhibition. The *crossed black lines* indicate loss of enzyme function in both directions

From what has been mentioned so far, the most likely model is as follows (see Fig. 2a, b): substitution of two arginine residues on both monomers inactivates both forward oxidative decarboxylation and reverse reductive carboxylation reactions while the presence of one arginine fully inhibits the forward oxidative decarboxylation reaction but changes the product of the reverse reductive carboxylation reaction to be (D)-2HG instead of ICT. In summary, it seems that the Mut-Mut dimer is totally inactive (i.e. does not interconvert ICT and 2KG and does not increase (D)-2HG production) while the Wt-Mut dimer does not interconvert ICT and 2KG, but increases the production of (D)-2HG from 2KG through the reverse reaction. Since (D)-2HG is thought to inactivate 2KG utilizing enzymes (i.e. because of its structural similarity to 2KG) [100, 101], it is possible that it also inhibits the Wt-Wt dimer form of the respective IDH isoform and that might explain the dominant negative effect of heterozygous arginine substitution (Fig. 2b) [56]. This model also explains why IDH1 R132 or IDH2 R140 only exists in heterozygous state. According to this model; when the cancerous cell that harbors heterozygous IDH1 R132 harbors another R132 mutation on the wild-type allele, the only IDH1 dimer in that cell would be Mut-Mut (Fig. 3a, b). This cell will no longer produce the oncometabolite (D)-2HG and will, therefore, lose the drive to multiply and will undergo apoptosis.

Interestingly, this model also explains the mutual exclusivity of IDH1 R132 and IDH2 R172 mutations documented in some studies [42, 102, 103]. From what has been explained so far and from the fact that biochemical and knock down studies proved that both IDH1 and IDH2 isoforms function interchangeably to support reductive carboxylation [31], it is possible that if a tumor cell happened to harbor both neomorphic alleles of the two isoforms (i.e. the tumor cell has both IDH1 R132 and IDH2 R172 neomorphic alleles), that tumor cell will lose its ability to synthesize enough citrate through the reverse reductive carboxylation reaction to keep up with its division and eventually dies. That would be due to the simultaneous and dramatic decrease in IDH1 and 2 enzyme reductive carboxylation activities and would also be due to the inhibitory effect of the excessively produced (D)-2HG on the relatively few IDH1 and 2 Wt-Wt dimers. A heterozygous mutation in one of them, on the other hand, will make the cell produce the oncometabolite, (D)-2HG and at the same time allow this particular (D)-2HG-producing tumor cell to produce enough citrate (i.e. through the other wild type isoform (e.g. wild type IDH2 in a tumor cell harboring neomorphic f IDH1) and utilize it to survive and divide. It is worth mentioning here that rare cases of double neomorphic IDH1/IDH2 allele AML cases have been reported. However, it is not clear whether these two neomorphic alleles were truly present in the same leukemic subclone [104]. Based on what has been explained so far, it is very likely that those two neomorphic alleles were present separately in separate subclones. Their simultaneous presence in those cases could have been an unavoidable artefact where the cellular DNA content of these separate clones could have mixed during DNA extraction that is upstream of mutation detection.

### Are somatic IDH1 R132 and IDH2 R172 activating mutations driver ones?

IDH1 R132 activating mutations were found by several sequencing studies to be very common in specific types of adult brain tumors, occurring in >70% of adult grade II and grade III gliomas and >80% of adult secondary glioblastoma multiforme (GBM) [105, 106]. It was also found that many of the grade II/III gliomas and secondary GBMs that are IDH1 wild type had mutations at IDH2 R172. Altogether, 80–90% of adult grade II/III gliomas and secondary GBMs harbor mutations at either R132 of IDH1 or R172 of IDH2. However, IDH activating mutations are present in <10% of primary GBMs and pediatric GBMs [42, 102].

Sequencing studies also confirmed that IDH activating mutations are frequent in clonal myeloid disorders and present in 5–20% of de novo normal karyotype acute myeloid leukemia (NK-AML) cases and in 10–20% of cases of secondary AML resulting from leukemic transformation of myelodysplastic syndrome (MDS) and myeloproliferative neoplasm (MPN) [107–112]. At lower frequency (5–10%), these IDH activating mutations are also present in chronic-phase MDS and MPN but are rare in translocation-positive AML [113–115]. In 10–40% of angioimmunoblastic T-cell lymphoma (AITL) were found to harbor IDH2 activating mutations, however these mutations were uncommon in other T- or B-cell lymphoid malignancies [115, 116].

Besides glioma, IDH activating mutations have been found in a number of other solid tumors. Over 50% of chondrosarcomas harbor IDH activating mutations [117–119], and IDH activating mutations have been linked to the pathogenesis of the enchondromatosis syndromes Ollier disease and Maffucci syndrome [120]. These are nonhereditary pediatric cancer predisposition syndromes. Children affected with these syndromes develop cartilaginous tumors when their developing bones undergo endochondral ossification. Interestingly, 40–90% of the tumors in these patients harbor mutations at R132 of IDH1 or R172 of IDH2 while having wild type IDH1 and IDH2 in their normal tissues, suggesting that these IDH activating mutations occurred as a postzygotic event and present in these patients as somatic mosaicism. It is worth mentioning that these patients are also at increased risk of other neoplasms, in particular, gliomas and AML [121, 122].

Around 10–20% of cholangiocarcinomas cases were found to have IDH activating mutations. Few cases of paraganglioma, colon cancer, prostate cancer, and lung cancer have also been found to harbor IDH activating mutations [43, 98, 123–126]. It is clear from these cumulative data that these IDH activating mutations are driver ones, which again emphasizes the importance of targeting them in these types of tumor.

### Are somatic IDH1 R132 and IDH2 R172 activating mutations early ones?

My hypothesis on this regard is that yes, these activating mutations are early ones in certain tumors and late in others. My elaboration on that is as follows: mutations can occur by chance in dividing cells due to replication errors or none dividing ones by several mechanisms including the exposure of cellular DNA to ROS, for instance. Notably, most common IDH1 and 2 activating mutations are caused by G>A or C>T transitions [e.g. R132H (CGT>CAT), R132C (CGT>TGT), and R172K (AGG>AAG)]. These transitions where G-C pair is substituted by A-T pair can be a result of cytosine deamination-a relatively common event-that escaped base excision repair. So, my hypothesis is that it is possible that these IDH activating mutations can occur by chance as early DNA variants in different cell types with different carcinogenic potential. In other words, it is possible that these substitutions have the highest carcinogenic effect (and might require only few cooperating mutations, if ever) when they occur by chance as an early event in a cell type that significantly relies on the reductive carboxylation activity of these two enzymes in its metabolism. Based on what has been mentioned earlier, it is likely that those cells that are normally active in producing citrate from 2KG are the ones that would be affected the most by these activating mutations. In these cells the presence of IDH activating mutation alone (without the presence of initiating or other cooperating mutations) would be enough to produce the oncometabolite (D)-2HG and therefore transform the cell into cancerous one. These IDH activating mutations in these tissues are early occurring ones (i.e. early driver mutations). In another tissue type (i.e. that is normally not active in citrate synthesis) the same activating mutation alone might not be carcinogenic and must be preceded by cancer initiating mutations causing this cell to start being active in citrate synthesis for cellular growth and division (see the previous section, “Their role in physiology and cancer”). Here, these IDH activating mutations are late-occurring ones, but still considered driver ones sine their presence “drove” the cell further towards being cancerous by e.g. DNA hypermethylation that will silence genes that are important for differentiation.

Interestingly, astrocytes (glioma cell of origin) and myeloid cells (from which acute myeloid leukemia develops) are known as high citrate synthesizer compared to the other tissue types. It is estimated that the citrate production and release from astrocytes is approximately 70 nmol/h/mg of protein [127]. That probably explains the high frequency of IDH1 and 2 activating mutations in neoplasms originating from these types of tissues [65] and agrees with my aforementioned hypothesis.

This same hypothesis can also explain why these IDH1 R132 or IDH2 R172 mutations have not been discovered in any non-glial subtype brain tumors [42, 105, 128]. Since these non-glial tissues are not active in citrate synthesis, the presence of IDH activating mutations alone might not have carcinogenic effect on that type of cell. To have neomorphic IDH harboring tumor originating from these non-glial cells (that are not citrate synthesizers), other tumor initiating mutations preceding IDH activating mutation must occur first, the probability of which is very low. Notably, prostate is also known to synthesize citrate in high amount [129] and R132 substitution has been found in prostatic cancer [43]. There are no known studies on the citrate synthesis in chondrocytes. However, chondrocytes are known to be active in synthesizing glycosaminoglycans [130]. Acetyl CoA is needed in these cells to acetylate the amino sugar glucosamine-6-phosphate, the building block of glycosaminoglycans [131]. The fact that 50% of chondrosarcomas harbor IDH1or 2 activating mutations, points to the high possibility that chondrocytes are active in citrate synthesis as a source of acetyl CoA and according to my hypothesis, IDH activating mutations are very likely to be early ones in this type of tumor. Interestingly, Hif1α has been shown to be an important surviving factor for chondrocytes [132]. So, it is possible that in chondrocytes, citrate synthesis is active and neomorphic IDH enzyme produces (D)-2HG (i.e. instead of ICT that would be converted to citrate). This oncometabolite as discussed earlier is known to inhibit Hif1α degradation and its excessive accumulation could be responsible for transforming chondrocytes into chondrosarcoma.

It is worth mentioning here that the compiled evidence suggests that in glioma, IDH1 activating mutation is often the first mutation to occur in that tumor [133, 134]. However; up to my knowledge, no one has come up with a hypothesis to explain that like the one I presented in this article.

From what has been mentioned so far, it is clear that IDH1 R132 and IDH2 R172 mutations in these types of tumor, whether they occur as early or late ones, are driver mutations and that stresses on the importance of targeting them as a candidate anticancer therapy. Based on my hypothesis, that targeted therapy will have beneficial effect on tumors harboring these mutations; especially those e.g. glioma and AML, where these mutations occur as early ones. In fact, drugs targeting IDH1R132 and IDH2 R172 mutant alleles have already been developed and their efficacy has been demonstrated by preclinical studies [67, 68, 135]. It is worth mentioning here that it is also plausible to study the efficacy of augmenting such treatment with glutaminase inhibitor, an inhibitor of glutamate transaminase such as aminooxyacetate, or asparaginases. Asparaginases are known to decrease plasma levels of asparagine and glutamine. They are approved by the US Food and Drug Administration for the treatment of acute lymphoblastic leukemia and have been among the most effective antileukemic drugs for treatment of childhood and adult acute lymphoblastic leukemia since the 1970s [136, 137].

Aminooxyacetate is a general inhibitor of pyridoxal phosphate–dependent enzymes, including transaminases. That means that in addition to decreasing 2KG, it would also deplete the amino acid pool within tumor cell and that in turn would impair its growth. Aminooxyacetate antitumor effect has also been proven in some preclinical studies [138–140]. It was also shown in other clinical trials to be tolerable at a dose of 1–2 mg/kg/day [141–143].

Notably, in vitro studies have proven the antitumor efficacy of glutaminase inhibitor on IDH mutant cells [144, 145]. It would be interesting to conduct a study where mutant IDH targeted therapy is combined with this drug (or any of the aforementioned drugs) to study their combined effect on IDH mutant tumors.

To our knowledge, results of such studies have not so far been published, probably because these studies have not been conducted yet. Such combined therapy might decrease (D)-2HG level dramatically by both inhibiting the mutant enzyme and limiting its substrate, 2KG.

It is worth mentioning here that, theoretically, decreasing intracellular 2KG level might have a deleterious effect on the 2KG requiring dioxygenases that are already inhibited in IDH1 R132 and IDH2 R172 mutant cancer. Theoretically, that might promote cancer growth. However, it is a matter of which is more sensitive to 2KG depletion, the physiological function of the 2KG dependent dioxygenases or the oncometabolite production by mutant IDH (i.e. IDH1 R132 or IDH2 172). Based on the in vitro studies on IDH mutant cells that have proven the antitumor efficacy of glutaminase inhibitor [144, 145], it is very likely that the beneficial effect of 2KG depletion on mutant IDH enzymes outweighs its unwanted effect on 2KG dependent dioxygenases.

Arguably, 2KG depletion might also have the unwanted generalized inhibitory effect on these dioxygenases present in normal cells. However, due to “oncogene addiction” phenomenon known for oncogene-dependent tumors, the effect of lowering 2KG on the tumor cells would be more dramatic compared to the normal ones, a concept proven by in vitro study [144].

Whether these drugs will cross the blood brain barrier to be taken up by neomorphic IDH harboring glioma cell is a question that further studies would answer. If they did work in combination with neomorphic IDH targeting therapy but failed to cross the blood brain barrier, then they would at least be considered for the treatment of other neomorphic IDH harboring tumors like myeloid neoplasms and chondrosarcoma.

When considering treatment modalities, one cannot underestimate the importance of oligonucleotides-based therapy e.g. allele-specific gene silencing by small interfering RNA (siRNA). Oligonucleotides are a promising new class of therapeutics that has been experimented in treating cancer and other disorders [146, 147]. There are still some challenges to overcome before their clinical application, including their rapid degradation, poor cellular uptake and off target effects. It is worth mentioning here that utilizing such therapeutic modality to treat inherited IDH disorders and IDH mutant cancer sounds like a feasible subject for future studies. Interestingly, the specificity of oligonucleotides towards the mutant IDH allele might not be of great importance. According to our model, silencing the wild type allele would be beneficial too, since that would also decrease the Wt-Mut dimer which is the (D)-2HG-producing one. Theoretically, silencing the wild type allele due to oligonucleotides off target effect would be of limited effect on tissues expressing both genes i.e. *IDH1* and *IDH2*. In these tissues, the normally expressed isoform (e.g. IDH1) will compensate for the loss of expression of the silenced one (e.g. IDH2). However, caution should be exercised when silencing the preferentially expressed IDH isoform in a particular tissue type e.g. silencing IDH2 in T cells to treat AITL (see below).

Lastly, some studies have confirmed that *IDH1* mutated cancer cells have significantly low intracellular level of coenzyme NAD+. That has been attributed to the low expression of NAD+ salvage pathway key enzymes. This metabolic vulnerability has been exploited as therapeutic modality where depletion of NAD+ using small molecule inhibitors targeting the salvage NAD+ synthesis enzyme nicotinamide phosphoribosyl transferase resulted in strikingly selective cytotoxicity in *IDH1* mutant cancer cells [148]. Interestingly, NAD+ is an essential donor molecule for poly (ADP-ribose) polymerase (PARP)-mediated single strand DNA repair. Its insufficient level in *IDH1* mutated cancer cells, decreased the efficiency of this important DNA repair mechanism in them and made them vulnerable to DNA-damaging agents. Based on that, the combined treatment of these cancer cells with olaparib, an inhibitor of PARP-mediated single strand DNA repair, and the DNA-damaging agent temozolomide has been proposed as a promising treatment modality for *IDH1* mutated tumors [149].

### The differential gene expression of IDH1 and 2

In general, due to differential gene expression, different tissues can have different active pathways. It has been shown that there is tissue-specific IDH2 expression in heart, muscles and lymphocytes. Interestingly, T cells-from which AITL originate- are known to express IDH2 in both resting and activated state with undetectable expression of IDH1 [150]. It is possible that in most tissues, excluding the aforementioned examples, IDH1 is the preferentially expressed IDH isoform. This could explain why IDH1 R132 germline mutation is compatible with life only as a mosaic form while IDH2 germline mutation is compatible with life in both mosaic and constitutional forms [151]. It could also explain why in most tumor types neomorphic IDH1 alleles are more common than those of IDH2, except in AITL where neomorphic IDH1 alleles has not been reported and AML where neomorphic IDH1 and IDH2 alleles have similar frequencies [104, 114, 115, 152, 153].

In T cells, IDH2-catalyzed reaction is more active than that of IDH1. According to my hypothesis, in these cells, the early occurrence of R172 (or less frequently IDH2 R140) mutations even, as lone one, will be enough to produce significantly high level of intracellular (D)-2HG and cause the cancerous transformation of that type of cell. Stated differently and based on the same hypothesis, it is likely that the lone occurrence of IDH1 R132 mutation in T cells would not make them transform into AITL. That is because (D)-2HG production would not be high enough to cause the cancerous transformation since IDH1-catalyzed reaction is not active enough in this type of cells. It is worth mentioning here that both rapamycin and cyclosporine A have been found to suppress IDH2 gene expression in T cells [150]. It would be interesting to study the benefit of adding one of these immunosuppressants to the combination therapy of AITL, especially IDH2-mutant type. In the case of AML, it is possible that in myeloid cells both IDH1 and IDH2 are equally active where the occurrence of IDH1 R132 or IDH2 R172 will produce the oncometabolite (D)-2HG in a cellular concentration that is high enough to cause them to transform into AML.

This differential expression of IDH1 and 2 in different tissues has important diagnostic and therapeutic implications and further complicates the task of not only differentiating driver mutations from passenger ones and early mutations from late ones, but also complicates the task of predicting which IDH mutant tumor would respond the most to mutant IDH targeting therapy. In other words; in this era of high throughput sequencing, finding IDH2 R172, for instance, in a tumor does not necessary mean that it is and early nor a driver one. Unless that mutation is too frequent to occur by simple coincidence in that type of tumor, labeling IDH1 R132 or IDH2 R172 mutations as driver ones in a tumor not frequently seen to harbor them and without proving the high content of (D)-2HG in that tumor, should be advised against. That is because it is possible that in that particular tumor the driver mutation was in another gene and IDH2 R172, for instance, was simply a passenger mutation in a gene (i.e. *IDH2*) coding for an enzyme the reaction it catalyzes was not active enough to produce enough amount of the oncometabolite, (D)-2HG. In fact, this precaution is not limited to IDH1 R132 and IDH2 R172 mutations, but should be borne in mind whenever we try to sort driver mutations from passenger ones in any type of tumor.

## Conclusions

Due to the structure of IDH3 and the unidirectional feature of its action, mutations in its coding genes would be of inactivating type that would have no cancer transforming potential. On the contrary, heterozygous mutations resulting in substitution of the key active site arginine cause IDH1 R132 and IDH2 R172 to gain a new function. These mutant enzymes lose the ability to catalyze the interconversion between ICT and 2KG. However, due to their newly gained function they produce the oncometabolite (D) 2-HG from 2KG. A model based on the ability of this metabolite to inhibit the Wt allele, explains the dominant negative effect and the mutual exclusivity known for IDH1 R132 and IDH2 R172 mutant alleles. This oncometabolite seem to explain IDH1 R132 and IDH2 R172 carcinogenic effect. It is possible that the more active the cell in converting 2KG to ICT, the more likely IDH1 R132 or IDH2 R172 (depending on the preferential IDH gene expression) mutations are early and driver ones. Based on that; in certain tumors, IDH1 R132 and IDH2 R172 mutations are considered as driver ones, emphasizing the importance of targeting them as a cancer treatment therapy in those tumors. Based on the hypothesis presented in this article; in certain tumors, these driver mutations are early ones and their targeted therapy will theoretically have the most beneficial effect. Preclinical trials have proven the efficacy of mutant IDH1 R132 and IDH2 R172 targeting therapy and combining them with 2KG lowering agents sounds like a feasible subject for future studies.

## Abbreviations

AITL: angioimmunoblastic T cell lymphoma
AML: acute myeloid leukemia
ccRCC: clear-cell renal cell carcinoma
(D)-2HG: (d)-2-hydroxyglutarate
D2HGA2: type II d-hydroxyglutaric aciduria
D2HGD: (d)-2-hydroxyglutarate dehydrogenase enzyme
ETC: electron transport chain
FAs: fatty acids
FH: fumarate hydratase
GBM: glioblastoma multiforme
Hif1α: hypoxia induced factor 1 alfa
HLRCC: hereditary leiomyomatosis and renal clear cell cancer
ICT: isocitrate
IDH: isocitrate dehydrogenase
(L)-2HG: (l)-2-hydroxyglutarate
L2HGD: (l)-2-hydroxyglutarate dehydrogenase enzyme
NK-AML: normal karyotype acute myeloid leukemia
PARP: poly (ADP-ribose) polymerase
PHD: prolyl hydroxylase
PHGDH: phosphoglycerate dehydrogenase enzyme
SDH: succinate dehydrogenase
TCA: tricarboxylic acid cycle
TET2: ten-eleven-translocation 2
VHL: Von Hippel–Lindau syndrome
MDS: myelodysplastic syndrome
MPN: myeloproliferative neoplasm
Mut: mutant
Wt: wild type
2KG: 2-ketoglutarate
